# The Emergence and Unfolding of Telemonitoring Practices in Different Healthcare Organizations

**DOI:** 10.3390/ijerph15010061

**Published:** 2018-01-03

**Authors:** Jannie Kristine Bang Christensen

**Affiliations:** Department of Sociology and Social Work, Center of Organization, Management and Administration, Aalborg University, Kroghstraede 7, 9220 Aalborg, Denmark; jbc@socsci.aau.dk; Tel.: +45-9940-2830

**Keywords:** telemedicine, home telemonitoring, practice-based approach, inter-organizational, health technology, healthcare system, technology adoption, emerging practice

## Abstract

Telemonitoring, a sub-category of telemedicine, is promoted as a solution to meet the challenges in Western healthcare systems in terms of an increasing population of people with chronic conditions and fragmentation issues. Recent findings from large-scale telemonitoring programs reveal that these promises are difficult to meet in complex real-life settings which may be explained by concentrating on the practices that emerge when telemonitoring is used to treat patients with chronic conditions. This paper explores the emergence and unfolding of telemonitoring practices in relation to a large-scale, inter-organizational home telemonitoring program which involved 5 local health centers, 10 district nurse units, four hospitals, and 225 general practice clinics in Denmark. Twenty-eight interviews and 28 h of observations of health professionals and administrative staff were conducted over a 12-month period from 2014 to 2015. This study’s findings reveal how telemonitoring practices emerged and unfolded differently among various healthcare organizations. This study suggests that the emergence and unfolding of novel practices is the result of complex interplay between existing work practices, alterations of core tasks, inscriptions in the technology, and the power to either adopt or ignore such novel practices. The study enhances our understanding of how novel technology like telemonitoring impacts various types of healthcare organizations when implemented in a complex inter-organizational context.

## 1. Introduction

Most Western healthcare systems face challenges in regard to changing demographics, with an increased population of elderly people with chronic conditions, higher demands about patient-centeredness and quality of care, and restricted resources. *Telemonitoring*, which is a subset of telemedicine technology, is articulated as one of the answers to these challenges, since telemonitoring is believed to be an innovative way to deliver care efficiently in a way that further supports patient-centered care (see, e.g., [1]). This belief has led to extensive experimentation with various forms of home telemonitoring services throughout the world (see [2]). Even though multiple pilot studies have shown promising results in regard to telemonitoring’s cost-effectiveness and patient-related effects (e.g., [3]), recent results from large-scale studies indicate that these effects are difficult to replicate when implemented on a large scale as part of routine services [4,5,6]. As this study illustrates, an explanation of this diminished effectiveness in large-scale projects can be found by focusing on the emergence of telemonitoring practices and how these practices are (dis)connected to the existing practices of the various health professionals responsible for delivering the telemonitoring services. 

Prior studies have shown how the uptake of new technologies and innovations such as telemonitoring (and telemedicine more broadly) requires their alignment with existing clinical practices found that usage of telemonitoring differed among medical specialities (e.g., pulmonary medicine and radiology) depending on their use of physical examinations or specialized investigative techniques; the more dependent the clinicians were on such examinations or techniques, the less they perceived telemonitoring as an appropriate clinical tool and compatible in practice [7,8,9]. Despite Lehoux et al.’s [9] focus on various medical specialities, their study is limited to a hospital setting, and this reflects a general trend in telemonitoring studies [10]. The result of this trend in telemonitoring studies is that a limited number of studies have investigated telemonitoring in an inter-organizational setting with multiple and disconnected practices across organizations and professions (see for instance [11,12], for such studies). However, the alignment of telemonitoring practice most likely differs in inter-organizational settings compared to mono-organizational settings, particularly when the organizations diverge with regard to services and competencies. In such inter-organizational settings, it is unclear which kind of telemonitoring practice will emerge, whether this practice can be connected with the different existing practices, and whether telemonitoring can connect separate practices across organizations. So, our knowledge about the unfolding of telemonitoring practices in an inter-organizational setting is limited, even though telemonitoring is believed to connect healthcare providers in new ways and ultimately reduce fragmentation of care among different healthcare providers [13]. 

It is against this backdrop, that this paper explores telemonitoring from an inter-organizational perspective. More concretely, the paper investigates the emergence and unfolding of telemonitoring practice in relation to the implementation of a large-scale inter-organizational telemonitoring program, called TeleCare North, in the context of the Danish healthcare system. The telemonitoring program can be characterized as a home telemonitoring service for patients with chronic obstructive pulmonary disease (COPD) and was implemented in municipalities, hospitals, and general practice clinics. The research question for this paper is as follows: How does telemonitoring emerge and unfold, as practices in an inter-organizational setting with divergent healthcare providers? 

The paper contributes to the current stream of literature on telemonitoring by offering a detailed description of the unfolding of telemonitoring at three different kinds of healthcare organization. Particularly, this study sheds light on a telemonitoring program primarily operated by the municipalities, which contrasts with most prior studies that investigate telemonitoring in a hospital, and thus highly specialized, setting [10,14]. Moreover, this paper contributes with knowledge about the varying performance of telemonitoring technology in the different corners of the healthcare system. This heterogeneous performance is connected to the health professionals’ different positions, power, and medical authority that either forces them to alter their work or enables them to ignore telemonitoring. These findings help to explain the diminished effect of telemonitoring technology when implemented in a complex, inter-organizational setting without shared authority structures. In such an inter-organizational setting, the health professionals and organizations hold different levels of power and are mutually dependent on each other to deliver healthcare to patients with chronic conditions.

## 2. Telemonitoring as a Technology

Telemedicine services, defined by the use of information communication technology to deliver healthcare services over a distance, are growing at a rapid pace, and a variety of telemedicine services are penetrating contemporary healthcare organizations [1]. Telemedicine services cover two sub-categories: technology used for diagnostic processes and communication between health professionals (e.g., tele-radiology and tele-dermatology) and technology used between health professionals and patients. The latter includes home telemonitoring, and is also called “telecare”. Home telemonitoring brings care directly to patients’ homes and mostly targets people with chronic conditions, where it is used to prevent hospitalization, to improve patients’ feelings of safety, and to empower patients to manage their chronic conditions [15]. However, telemedicine, including telemonitoring, is more than a technological innovation. Rather, it can be perceived as a system of care [16] requiring actors who perform necessary functions in order to make the telemonitoring technology work [17]. This system includes for instance organizational change, the reconfiguration of existing relations and practices, and institutional change that supports the uptake and broader legitimacy of telemedicine [18]. Hence, telemedicine such as telemonitoring has various implications for existing diagnostic and clinical practice. The TeleCare North program and the related research was presented for the Regional Ethical Committee for Medical Research in the North Denmark Region where it was determined that noethical approval was necessary.

## 3. Telemonitoring as a Practice

A promising way to further our understanding of uptake of telemonitoring technology is to investigate the practices that emerge in relation to telemonitoring and how these emerging telemonitoring practices are (dis)connected to the existing practices of various health professionals from different organizations. Using a practice-based approach [19,20,21] involves activities, performativity, materiality, and work. Practices are, in the context of this paper, defined as:

Routinized types of behavior which consist of several elements, interconnected to one another: forms of bodily activities, forms of mental activities, ‘things’ and their use, a background knowledge in the form of understanding, know-how, states of emotion and motivational knowledge ([21], p. 249).

This definition makes obvious that practices cannot be reduced to activities and actions, since practices produce and reproduce meaning, identities, and ordering of the social. Therefore, practices entail knowledge, certain ways of understanding the world, norms, and expectations about how to behave and act appropriately in a given context, and practices thus reflect the historical and institutional context in which they are performed. This context offers both possibilities and constraints for the unfolding of practices. However, context is not external, nor comprised of superstructures that determine actors’ actions and behavior. Instead, social structures are recursively intertwined with practices and are in fact produced and reproduced by ongoing activities undertaken by actors [19,20]. 

Relatedly, organizations are continually made and re-made through the practices undertaken by organizational actors. From this perspective, organizations are viewed as bundles of practices connected in various ways through webs of relationships and mutual dependencies [20]. The same can be said of practices across organizations; they are also connected in complex ways, and these connections are enforced when the organizations and their members interact regularly and are interdependent on each other’s services or products. From this perspective, organizational practices are not restricted to internal organizational matters; instead, they are connected to other organizations’ practices through inter-organizational relationships, mutual dependencies, and joint activities. As a result, changes in one of the organization’s practices may lead to changes in the collaborating organizations’ practices, due to mutual adjustment. Such adjustments may be contingent on the different organizations’ and actors’ positions and power to resist or initiate change [22]. In situations where the established practices are disturbed, for example by the introduction of novel technology or treatments, such mutual adjustments are more apparent, and reconfigurations of existing relations may occur (see, e.g., [9,12,23]). 

On a practice-based approach, objects are given the same role as cognitive, discursive, and bodily components in practices, since the performance of a practice often involves using certain objects in a specific way [20,21]. Correspondingly, telemonitoring technology can be viewed as an object that constitutes an important component in understanding the emergence and unfolding of telemonitoring practice. As many others have argued, technologies are not neutral devices. Rather, they are inscribed with the designers’ intentions, expectations of use, and assumptions, which both constrain and enable different types of behavior and action. However, this does not mean that a technology is used as intended, since in their use of the technology users may be ‘ignoring certain properties of the technology, working around them, or inventing new ones that may go beyond or even contradict designers’ expectations and inscriptions’ ([24], p. 407). The practices that emerge are thus never ‘given’ by the design of the technology but are the result of the users’ work to make them meaningful and connect them to their existing practices and understandings of the world. Consequently, the same technology may perform rather differently in different social settings, since the performance depends on how users transform, adopt, ignore, or reject the technology [17,24,25]. 

From a practice-based approach, telemonitoring technology can be viewed as an innovative technology that has the potential to affect existing clinical practices and care practices within the healthcare system. However, the outcomes of telemonitoring are dependent on the specific organizational, institutional, and historical context, and much depends on users (i.e., in the present study, healthcare professionals) and how they make telemonitoring meaningful in relation to their existing practices. In an inter-organizational setting where divergent healthcare organizations jointly operate a telemonitoring program, the emergence and unfolding of a telemonitoring practice is likely to be influenced by their different positions and power to shape the unfolding of the practice, as illuminated in the remainder of this text. 

## 4. Case and Context

The program studied, TeleCare North, is a large-scale telemonitoring program in the north of Denmark and focuses on the home telemonitoring of COPD patients who self-measure oxygen level, blood pressure, pulse, and weight, and who answer questions about their symptoms. Home telemonitoring is a so-called store-and-forward system, where the patients’ data are transmitted to a shared monitoring database for the municipal actors, hospital staff, and general practitioners (GPs) (i.e., the health professionals and patients do not interact directly). The shared monitoring database contrasts with the conventional mono-organizational information technology (IT) systems that dominate the Danish healthcare system (e.g., electronic patient records at hospitals and electronic care records in municipalities). The design of the shared monitoring database is assumed to improve collaboration between municipalities, hospitals, and GPs by offering easy and fast data-sharing. 

The overall goals of TeleCare North were to improve treatment for COPD patients, reduce hospitalizations through prevention and a pro-active approach to the patients, reduce the length of admissions, reduce control visits at the hospitals, and to improve collaboration between municipalities, hospitals, and GPs [26]. Realization of these goals where conditioned by collaboration among the three main health care providers in Denmark (i.e., municipalities, hospitals, and GPs) as well as changes in the existing practices and approaches to COPD patients within each of these healthcare organizations.

**Figure 1:**
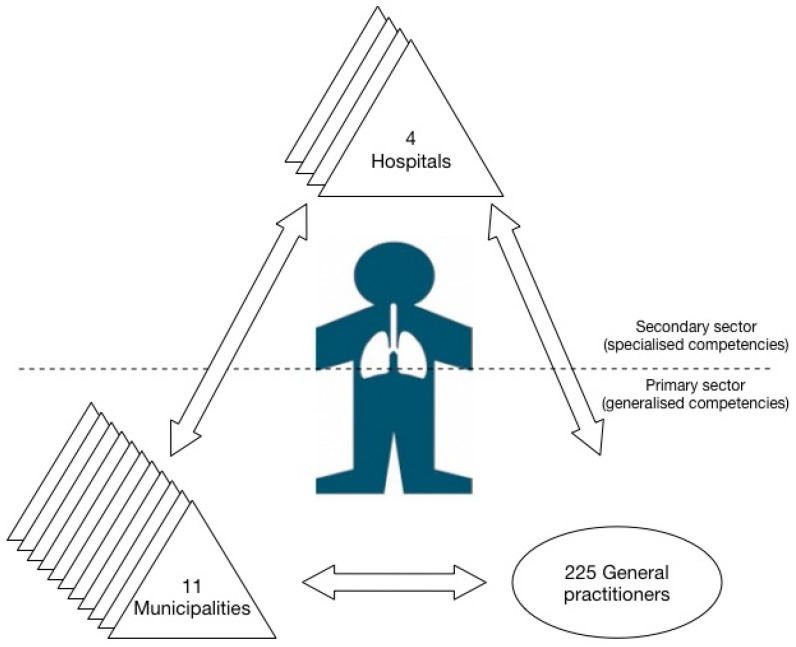
The telemonitoring network.

TeleCare North is operated in an inter-organizational setting involving the primary and secondary healthcare sector. The health care system in Denmark is primarily public and financed by taxes. The primary healthcare services are provided by the municipalities and self-employed GPs who function as gate keepers to the health system. Secondary healthcare services are provided by the hospitals led by the regions [27]. The program involves 11 municipalities (with five local health centers and 10 district nurse units participating in the program), four hospitals (with lung wards and outpatient clinics), and 225 GPs (see Figure 1).

Municipalities are the main actors in the program, since they monitor patients with less severe COPD or patients with stable conditions, which characterize the majority of the 1225 enrolled patients. This paramount role contrasts with their minor role in conventional treatment of COPD patients, where municipal health centers offer rehabilitation programs to train patients (both physically and mentally) to live with their chronic conditions. Moreover, the municipal district nurse units provide homecare services and daily living assistance to the patients with the most severe COPD. Although municipalities, like any other organization, consist of bundles of practices, the overall practice is a care-practice with a focus on care, non-acute work, generalist knowledge, and a holistic approach to the patients. This practice is also reflected in the organization of the work, which occurs mainly in day-time shifts. In the municipalities, the health professionals mostly consist of nurses, nursing assistants, and physiotherapists without medical authority or responsibility for the treatment of patients, although treatment can be delegated to the nurses by the GPs or the physicians at the hospitals. Accordingly, the municipal nurses are often dependent on the GPs or hospital physicians in relation to treatment of patients. This dependency of medical authority is also visible in the practices in the municipalities where collaboration and coordination of activities with GPs and hospital staff often is part of the every-day routines.

Together with the municipalities, the GPs are the main actors in the primary healthcare sector. The GPs function as ‘family doctors’, and in the conventional treatment of the COPD patients, the GPs are responsible for their treatment, regular control checks, and referral to specialized treatment or municipal health services, when necessary. Although the GPs’ practice can be characterized as a medical practice with a focus on treatment and medical issues, their work is also characterized by being non-acute, using generalist medical knowledge, and relying on a holistic approach to the patients. They are, in contrast to the municipal health professionals, able to work relatively autonomously, with a high degree of professional discretion. Their role in the telemonitoring program is to enroll patients, provide medical assistant to the municipal actors (e.g., by adjusting threshold values in the monitoring database, adjusting treatment based on the municipal nurses’ assessments of the patients’ data, and discussing patients’ treatment with the municipal nurses in complicated cases), and maintain their role as family doctors. Moreover, the GPs were expected to use the patients’ data in the monitoring database in their COPD-related consultations with the patients or to plan regular control checks. So based on the program the GPs were expected to intensify their collaboration with the municipal actors and change their practice in relation to their contacts and consultations with the patients.

The hospitals are the main actors in the secondary healthcare sector, and they offer specialized diagnosis and treatment. In conventional treatment of COPD patients, their role is to treat the most complex and severe patients in relation to hospitalizations, outpatient-clinic visits, specialized treatment, and specialized diagnostic methods. Their work in relation to COPD patients focuses on treatment, acute work, ‘hard evidence’ such as vital signs, and specialized knowledge reflecting clinical practice. In the telemonitoring program, the hospitals’ role was to monitor the most complex and ill patients in the program and use telemonitoring to treat the patients more effectively. For instance, they were supposed to replace control visits with telemonitoring. Moreover, when the patients were monitored by the municipal actors (which were the majority of the patients), the hospital staff could use the municipal actors knowledge about the patients to plan control visits (e.g., postponing control visits for patients in stable courses). To realize the goals of TeleCare North it was important that the hospital actors changed their existing practices and integrated telemonitoring into their work to replace some of the existing activities in relation to treatment of COPD patients. This change in practice was partly conditioned by internal factors (e.g., aligning with medical norms) and external factors such as collaboration with the municipal actors who would often monitor the patients before and after hospitalization of the patients. Table 1 summarizes the characteristics of the divergent approaches to treatment and care of COPD patients among the municipalities, hospitals, and GPs.

As this short description reveals, the three divergent healthcare providers are mutually dependent on each other to treat patients with COPD sufficiently. However, treatment of this group of patients has been criticized as fragmented across the three healthcare providers, which motivated the involved organizations to develop a shared telemonitoring program. As a result of extensive collaboration on the shared program, the telemonitoring program resembles a mix of a clinical practice with a focus on core vital signs and municipal practice in regard to the operation of the program where tasks were performed to fit into a municipal organization of activities (e.g., according to the day-time shifts work routines) (for more see, [28,29]).

## 5. Design and Methods

From 2012 to 2015, a longitudinal qualitative case study was conducted to obtain knowledge about how telemonitoring practices emerge within and between various health provider organizations. The case was the inter-organizational telemonitoring program termed TeleCare North, located in Denmark, which involved novel practices unfolding in inter-organizational settings. This study comprised one part of that longitudinal study and covers a period of 12 months, from February 2014 to February 2015.

Health professionals from various municipalities’ health centers, district nurse units, hospital wards, outpatient clinics, and general practice clinics were interviewed and observed by the author. These health professionals were responsible for handling the various telemonitoring tasks at the operational level (e.g., monitoring of patients, instruction of patients, or adjustment of treatment based on data generated from the patients home monitoring). The local project managers who were responsible for implementing the program in their local organization helped to identify the health professionals who were formally assigned to work with telemonitoring and furthermore they helped to establish a contact to these health professionals, except from the GPs who were recruited through a direct contact. As a result, five municipal nurses, two hospital nurses, two lung physicians, and six GPs participated in this study (see Table 2).

### 5.1. Interviews

The health professionals were interviewed twice, with the first round conducted six months after implementation of the program, and the second a year-and-a-half after implementation. The interviews were semi-structured, single, interviews that lasted from 30 to 120 min and were conducted by the author. Most interviews were conducted face-to-face at the health professionals’ workplace, except from the interviews with the GPs which were conducted by phone due to practical issues (e.g., most of the GPs preferred to call whenever they had a break in consultations instead of making a physical appointment for the interview). All interviews were recorded and fully transcribed.

The interview-guide was constructed to investigate how telemonitoring materialized within the different organizations and was utilized by the various health professionals in relation to their daily work, existing practices, and their relationships with health professionals from the other healthcare providers. Correspondingly, the interview guide included themes concerning the use of telemonitoring, changes in work routines and in inter-organizational relations, challenges in the use of telemonitoring, and the organization of the telemonitoring tasks. These themes were constructed with inspiration from Nicolini [17] but also resembled a more explorative approach to studying the unfolding of telemonitoring in established work practices. The questions were open-ended to give the health professionals opportunity to articulate other aspects and bring in new perspectives which corresponded with the explorative approach.

### 5.2. Observations

To get a more thorough understanding, observation studies of the health professionals were conducted. These studies concerned the municipal and hospital nurses, since they were responsible for monitoring the patients. The nurses were observed when they performed the telemonitoring tasks and were conducted in relation to the interview (see Table 2). During the observations the mundane, unreflective and hidden activities and work were illuminated, supplementing the interviews [30]. 

Additional observations from four meetings in a group of local project managers in the program were made. The group consisted of 11 municipal project managers (i.e., one from each municipality) and one regional project manager who represented the four hospitals. The local project managers were responsible for implementation of TeleCare North in their organization and represented different professional backgrounds (some had a health professional background whereas others were administrative workers). The aim of this group was to facilitate a relatively uniform implementation of the program in the divergent organizations. Moreover, one meeting in a group of health professionals (GPs, lung physicians, hospital nurses, and municipal nurses) in the program was observed. The aim of the group was to develop the health related content in the program according to existing professional standards and guidelines (see Table 2).

In relation to these observation studies detailed notes were written about actions, behavior, interactions, content of conversations and direct citations from conversations, disagreements, relational aspects, and context. Particularly, the interactions, disagreements, and relational aspects were important in the observations of the group meetings whereas the focus of observations of the health professionals centered around their practical use of the telemonitoring technology and our informal conversations about telemonitoring. All observations were conducted by the author.

### 5.3. Document Studies

Lastly, various documents were studied to get a more comprehensive understanding of the program and the different inscriptions in the program. Descriptions of tasks, roles, functions, divisions of responsibilities, and work instructions were among the documents studied, as well as minutes from meetings, the business case for the program, local and national healthcare strategies, and the like (see Table 2). A total of 28 semi-structured interviews, 10 h of observation of health professionals, 18 h of observation of the project managers’ meetings, and various documents constituted the empirical data collected in this study.

### 5.4. Data Analysis

The data analysis was performed in different steps. First, the data were coded by the author in NVivo (version 10, 2012, QSR International, Doncaster, Australia) (software program for organizing qualitative data). Various codes were constructed on the basis of the themes in the interview guide (e.g., use of telemonitoring, change in work routines and practices). Data were coded to display principles associated with the various practices in the municipal health care centers, district nurse units, hospital wards and outpatient clinics, and at the GP clinics. Next, data were coded to identify changes over time, with particular attention to alignment and change of work practices. More specifically, the empirical data from the two interview rounds was read and coded comparatively to identify changes to how telemonitoring was used by the different health professionals. The analysis was performed without an explicit theoretical framework (although inspired by Nicolini [17,20]), as reflected in the presentation of the empirical findings. The analysis focused on finding patterns in how telemonitoring was used and (not) connected to existing practices and norms. In this analysis it was obvious that telemonitoring was used rather differently by the actors and that this use was conditioned by the organizational and professional affiliation which is reflected in the findings that are themed according to organizational affiliation. Finally, data extracts (i.e., quotes and observation notes) are translated from Danish to English in the context of this paper.

## 6. Findings

### Emerging Telemonitoring Practice

The core tasks in the telemonitoring program involved enrolling patients; training the patients to use the electronic equipment; educating them about COPD to empower them to manage and live with their disease; and assessing the patients’ data and reacting to the worsening of their disease to prevent exacerbations and hospitalizations (e.g., by calling the patient or the patient’s GP, initiating or adjusting treatment). Formalized work descriptions, roles, and the division of tasks and responsibilities regulated and structured the various activities related to the core tasks into sequenced orders. Moreover, the monitoring database was designed to support decision-making on how to react properly to the patients’ measurements since alarms were activated depending on whether the measurements surpassed pre-defined thresholds. These various elements shaped the emerging telemonitoring practice and the cognitive, normative, and behavioral components of that practice.

Accordingly, telemonitoring involved new forms of activities related to the core tasks in the program, but it also required another way of understanding what constitutes good clinical practice in regard to assessment of the patients’ conditions; identification of exacerbations; initiating, adjusting, and terminating treatment; and more relational aspects, such as interpersonal relations with the patients and more extensive collaboration with other health professionals. As such, practices also involved routinized bodily actions reflecting routinized ways of understanding the world and knowing how to do something [21]. The telemonitoring tasks implied other bodily performances compared to the activities related to existing practices, where explicit physical examination (respiration, inhalation techniques, etc.) and more tacit physical examination (e.g., like the patients’ appearance, their surroundings, and interactions with relatives) were perceived as important. However, telemonitoring altered these activities since the health professional and the patient became separated in time and space; the health professionals could not form a holistic impression of the patient and his or her condition, but had to rely on patients’ own (objective) measurements of vital signs and their (subjective) assessments of their symptoms (for similar findings, see also Lehoux et al. [9]). Accordingly, telemonitoring altered both the core tasks, routinized bodily performances, and challenged the established understandings and ways of dealing with the COPD patients. 

To address these changes, it was necessary to associate norms, values, and meanings with telemonitoring. A great part of this work was done prior to the implementation of the program, where the specific content of the program was developed by different health professionals and administrative workers from the different organizations in the telemonitoring network. In this work, telemonitoring was sought, aligned with existing perceptions of ‘best practices’ for treating patients with COPD (for more, see Christensen [28]). Furthermore, various strategies were undertaken to legitimize telemonitoring from different perspectives. However, these development activities and strategies for legitimation mostly reflected a discursive level, whereas meaning still needed to be established in relation to the day-to-day routines and mundane work in the municipalities, hospitals, and GP clinics. 

## 7. Aligning with Divergent Municipal Practices

Since the content of the telemonitoring program relied on clinical practice with heavy use of disease-specific knowledge and a focus on vital signs (i.e., a ‘hospital approach’), and since the main actors who operated the program were the municipal actors, different challenges arose when translating the program into practice. Telemonitoring required another way (i.e., a hospital ‘way’) of understanding assessment and treatment of the COPD patients. Interpreting the patients’ data from the telemonitoring database required that nurses had more specialized knowledge about COPD symptoms, and it involved a high degree of clinical judgment. Reacting properly to the patients’ data was perceived as difficult, since it only relied on the core data and not on a holistic assessment of the patient (e.g., based on the patient’s appearance that would normally be visible in a face-to-face interaction). Hence, telemonitoring reconfigured the involved health professionals’ practices by disembedding care and interaction with the patient in time and space. Surprisingly, reconfiguration of these practices did not consist of alignment or adoption of a hospital approach or clinical practice. Instead, a tentative telemonitoring practice was emerging in the municipalities, where focus on empowerment of the patients was at the core, along with a strong orientation towards vital signs. The focus on empowerment of the patients reflected a rehabilitation practice used in the health centers, where the core tasks in relation to COPD were rehabilitation programs where the patients exercised and gained knowledge about their disease and how to live with it. A municipal health center nurse explained:

*“We use the patients’ monitoring data in our conversations with the patients at our rehabilitation programs. We use it as a tool to talk with the patients (about their current condition) and use it as an integrated part of our rehabilitation work with these patients”*.

As she demonstrates, telemonitoring was given meaning through integration and alignment with existing practices. In that sense, telemonitoring was perceived as “*a perfect fit with the health centers’ rehabilitation programs*,” as another health center nurse stated. However, telemonitoring was not as easily connected to the practices in the municipal district nurses’ units that reflected a more traditional, paternal nursing logic and care-practice, where “*nurses are good at giving answers but not as good at letting the patients reflect themselves and come to the answers—you know to create patient empowerment*,” as the regional project manager explained. The change of approach to the patients caused by telemonitoring was recognized as a challenge for the district nurse units, as was apparent in a meeting in the implementation group.

The local project managers discuss how the district nurses have to learn to let go of the patients. Traditionally, the district nurses solve the problems for the patients and take care for them, but now they have to let the patient do it themselves and facilitate patient empowerment. This is something they are used to doing in the health centers in the municipalities but not at the district nurse units. (Observation notes, implementation group).

As these different examples demonstrate, telemonitoring was easily made meaningful and connected to the health centers’ existing practices, whereas telemonitoring involved a significant change of mindset and approach to the patients for the district nurses and was difficult to connect to their other tasks. As a result, telemonitoring was unfolding rather differently *within* the municipalities.

## 8. Missing Connection with GPs’ Practice

The telemonitoring concept in the program carried some inscribed instructions for actions visible in relation to the threshold values. General threshold values were set to a default condition for the patients, but the GPs or lung physicians could change them and individualize them to each patient if necessary. Whenever a measurement of the vital signs was outside the threshold values, an alarm signaled so in the monitoring database, prompting an assessment of the patient’s condition. However, this emphasis on threshold values and vital signs collided with the GPs’ individual approach, where prior knowledge about the patient’s reaction patterns and the personal relationship with the patient were more important when assessing the patient and deciding the right intervention, as exemplified by one GP.

We move away from our more individual-centered approach where we know each patient personally and how the patient reacts to his or her symptoms. Now in the telemonitoring program you have these threshold values (for the different vital signs) that define when to intervene instead of looking at the patient’s overall condition and using your knowledge about this patient’s normal reaction. Some patients are quick to react to changes, whereas others wait until the last minute, and then when they call, we know that something is really wrong (GP).

Aside from this narrow focus on vital signs, the inscribed expectations in the telemonitoring concept also collided with the GPs’ existing practices in another matter. The GPs were used to the patients coming to them when experiencing worsening in symptoms, seeking medical advice, and so on. However, the telemonitoring concept was designed such that the health professionals could react if the patients’ condition worsened. This approach differed completely from the GPs’ more reactive approach to patients, as was indicated:

*“So I expect and trust that the patients contact me if they need help—both in relation to TeleCare North and more generally in relation to how we GPs work; we don’t do outreach work. We sit here (at the clinic) and then the patients come to us”*.

As this explanation also demonstrates, this inscribed expectation about doing more outreach and working pro-actively did not affect how the GPs worked. Similarly, the GPs’ practice did not move away from their individualistic approach. As such, none of the GPs changed how they treated the patients, and a telemonitoring practice did not emerge among the GPs in the studied period. One explanation of this inflexibility is the GPs’ highly autonomous position in the medical profession [23] combined with the division of labor in the telemonitoring program hardly affecting their work; the GPs still had the power and position to continue their work as usual, unaffected by the telemonitoring program, and since the program neither directly benefitted the GPs nor removed any of their tasks, their motivation to use the program was rather low. However, this lacking emergence and recognition of telemonitoring as a viable way to access the patients had consequences. One of the goals of the telemonitoring program was to prevent hospitalizations through more pro-active approaches where worsening in the patients’ condition would be detected early and handled by the municipal actors and GPs. In some instances, though, this was difficult to realize since the GPs did not use telemonitoring or recognize it as suitable in relation to accessing the patients’ condition and this challenged collaboration between municipal actors and GPs. One example of this was noticed in relation to a worsening in the patient’s condition which was observed by the municipal nurses. Concretely, one of the municipal nurse observed a lower oxygen saturation in the patient’s blood via the monitoring database and contacted the GP to get the patient referred to more detailed diagnostic methods at the hospital. However, the GP did not recognize the monitoring data as valid enough to refer the patient but insisted on physical examination of the patient before he would refer the patient to the more detailed diagnostic methods at the hospital. One of the project managers referred to this instance at a meeting in the health group:

*“We have this instance where a district nurse (from the municipality) contacts the GP since one her patients has a low saturation and is clinical affected in terms of shortness of breath and bluish around the lips and nails. The district nurse’s clinical judgement is that the patients ought to be referred to a blood-gas analysis at the hospital (more advanced diagnostic method) which can be done by the GP. However, the GP refuse to refer the patient based on these monitoring data but insist on examining the patient himself in his clinic”*.

This example illustrates how the municipal nurses are dependent on the GPs in order to get the patients referred to specialized diagnostic methods and treatment. Moreover, this example illustrates how some of the GPs did not perceive telemonitoring as a valid way replacement for physical examinations. Lastly, the example illustrates the non-emergence of a telemonitoring practice at the GPs made it difficult for the municipal actors to connect their emerging telemonitoring practice with the GPs which challenged collaboration between them and constraint their ability to perform their telemonitoring tasks (e.g., in terms of preventing hospitalizations). Although several other municipal actors reported about difficulties in relation to the non-use of telemonitoring at the GPs, several positive instances in relation to collaboration with the GPs were also reported (for more see, Christensen [28]).

## 9. Lacking Utilization in a Hospital Setting

The existing work practices at the hospitals were relatively unaffected by telemonitoring. The obvious explanation for this lack of effect was that the hospitals monitored few patients. From the perspective of the hospitals, the enrolled patients were either too well to be monitored by the hospitals or too ill (i.e., needing palliative care instead of treatment) for the hospitals to have any role, despite a baseline measurement at the beginning of the program that showed that 56% of the enrolled patients had severe or very severe COPD (groups III and IV, according to the Global initiative for chronic Obstructive Lung Disease (GOLD) classification of severity) [31]. In the overall health agreements between the municipalities and the regions, the hospitals are primarily responsible for treating patients with severe or very severe COPD [32]. Although hospitals were expected to play a larger role in TeleCare North due to the baseline measurement and the health agreements, they had a marginal role in the program at the operational level, and telemonitoring was “*not at all integrated in (their) work*” (hospital nurse). However, another explanation of their marginal role was that the developed telemonitoring concept was perceived as difficult to utilize as a substitute for some of the conventional hospital activities in relation to treatment of patients with COPD (e.g., reducing length of hospitalizations by monitoring the patients at home or substituting some of the outpatient-clinic control visits with telemonitoring), although the content in the program reflected a hospital logic. Concretely, the lung physicians and the nurses at the hospitals did not perceive telemonitoring as appropriately designed to substitute existing activities. For instance, telemonitoring could not replace outpatient clinic control visits according to the regulations from the Danish Health Authority, as one of the lung physicians at a hospital explained:

*“The Danish Health Authority has some guidelines for control visits which, for instance, involve control of the patients’ inhalation technique (…) so you have all these different requirements to a control visit that telemonitoring cannot replace”*.

This example also demonstrates how the telemonitoring program was embedded in a highly institutionalized and regulated field constraining how much could be changed without violating national regulations and requirements for treatment. In another example, one of the hospital nurses explained how the patients’ monitoring data were misaligned with the knowledge needed in the outpatient clinics:

*“The way we work here at the outpatient clinic, well the decisions are based on a current measurement of the patient’s vital signs—for example, decisions about oxygen treatment—and not based on prior measurements (conducted by the patients at home with their telemonitoring equipment)”*.

From these two examples, it is obvious that telemonitoring was not given meaning as a clinical tool and as an appropriate replacement for the existing activities related to treatment of the COPD patients. This understanding of telemonitoring was supported by a discussion between some of the lung physicians about the use of telemonitoring when terminating oxygen treatment, as the following extract from my observation notes illustrates.

The lung physicians at the different hospitals disagree about what is perceived as sound professional practice in relation to telemonitoring. For instance, they disagree about whether oxygen treatment can be terminated by relying on telemonitoring or not. Some of the lung physicians fully support termination of oxygen treatment by the use of telemonitoring, whereas others totally reject that telemonitoring can replace physical controls. (Observation notes).

This disagreement was also reflected in the interviews with the two lung physicians, as one perceived telemonitoring to be an alternative to physical visits in relation to the termination of oxygen treatment, whereas the other believed that a physical visit remained necessary, since “*you know, there is a reason to be treated with oxygen—and that is the disease that causes it (COPD) and that (the disease) still needs to be checked properly (at a physical visit)*”. Accordingly, consensus about use of telemonitoring and alignment with existing professional norms were not obtained within the hospitals since some lung physicians believe that data from telemonitoring can be used to inform clinical practice whereas others totally disagree.

Although hospital staff was involved in developing the telemonitoring program, the program was, surprisingly, not perceived as useful in replacing activities in the outpatient clinics. Moreover, telemonitoring was perceived as not producing the ‘right’ form of knowledge for clinical practice, as the above example demonstrates. This perception led to the non-emergence of a telemonitoring practice at the hospitals, and telemonitoring was instead ignored.

## 10. Discussion

### 10.1. Power to Adopt, Modify, or Ignore Inscriptions in Telemonitoring Technology

This study contributes to research concerning technology uptake in organizations and the numerous empirical studies of telemonitoring by detailing the unfolding of telemonitoring technology in various organizational contexts and illuminating how telemonitoring performs rather differently in these settings, depending on the resilience of existing practices and the actors’ power to ignore telemonitoring [7,9,17]. Technology such as telemonitoring technology embodies the designers’ intentions, desires, and assumptions about the users and the work the technology is believed to perform [24]. The use of the technology, however, is not determined by such inscriptions, but is the result of ongoing negotiations between the technology and the existing work practices of the users, which may result in differential technological performance [17,25]. 

The telemonitoring program was loaded with various scripts that ordered the various core telemonitoring tasks in sequence and promoted a certain way of acting. Although the emerging telemonitoring practices to some extent reflected these inscriptions, the unfolding of the telemonitoring practices was rather different in the various healthcare organizations. The unfolding of telemonitoring practices varied depending on the health professionals’ role in the program, their ability to align telemonitoring with their own professional practice, and their power to adopt, adjust or ignore telemonitoring. At one pole, the municipal nurses were highly affected by telemonitoring, as they were the main actors in regard to monitoring the patients’ measurements. For the health centers, telemonitoring was easily adopted and adjusted to existing practices, whereas the district nurses struggled to align telemonitoring with their existing practices because it collided with their care practices and more traditional, paternalistic approaches to the patients. However, as the district nurses did not have medical authority or an advantageous position within the medical hierarchy (Abbott, 1988 [23]), they were forced to adjust their practice according to telemonitoring. 

At the other pole, the GPs and the hospital staff were relatively unaffected by telemonitoring. Since telemonitoring was perceived as a movement away from the GPs’ more individual-centered approach to patients and was perceived as a threat to their well-established professional constructs regarding their roles as family doctors, nearly no change in the GPs’ practices or approach to the patients was detected. The GPs continued ‘business as usual,’ unaffected by telemonitoring, a behavior enabled by their relatively withdrawn role in the program and their dominant position as a medical profession with a high degree of autonomy. Similarly, hospital staff were largely unaffected by telemonitoring, partly as a result of their marginalized role in the program and their position as medical specialists, which gave them the power and autonomy to continue their existing work relatively unaffected by telemonitoring. 

As this case illustrates, the processes of the emergence and unfolding of novel practices may be the result of a complex interplay between existing work practices, alterations of core tasks, inscriptions of the technology, positions in the professional system, and the power to either adopt or ignore such novel practices. More specifically, when the technology is not easily aligned with existing practices, ignoring the technology may be a possibility only when the actors have enough power to continue their work practices as usual. This explanation resonates with prior research on change in organizations dominated by strong professions, which has shown how professionals are able to resist change due to their high level of autonomy [33,34].

### 10.2. Connecting Practices across Divergent Organizations: Reproducing Power Inequalities

Since practices are connected in webs of relationships, joint activities, and dependencies, changes in practices at one site often produce changes and adjustments of practices at other sites. Through information communication technologies, such as telemonitoring technology, practices that are otherwise separated in time and space can be connected, related, or reconfigured in new ways [35]. Although this connectivity may seem promising in relation to integrating activities across divergent healthcare providers, it may have the reverse effect when telemonitoring is used rather differently. In this case, telemonitoring was silently ignored by two of the main healthcare providers, which influenced both the last set of actor’s (i.e., the municipalities’) ability to perform their telemonitoring tasks and their ability to connect their emerging telemonitoring practice to the others. As a consequence, the municipalities did not have enough support in terms of medical expertise to solve their core tasks of telemonitoring the patients’ data and had to find alternative strategies to gain this support (for more, see Christensen [28]). Since the municipal nurses were at a disadvantaged position in terms of power, due to their lower position in the professional system [23], they had to adjust their work practices to the GPs and hospitals’ work practices and uptake of telemonitoring. As a result of this varying uptake of telemonitoring, the effects of telemonitoring in reducing hospitalizations and replacing existing activities with telemonitoring were modest [6]. 

### 10.3. Limitations

It can be difficult to determine whether the observed activities are actually forming patterns of routinized activities or are merely expressions of sporadic, non-routinized activities. In this case the novel activities related to telemonitoring were studied over a period of 12 months, during which the (non) practices were crystallizing. At the beginning of the study, a telemonitoring practice was emerging in the municipalities, whereas it was non-observable at the hospitals and the GPs. Although the telemonitoring practice further crystallized and was routinized in the municipalities, it remained intangible among the hospitals and the GPs. Although the telemonitoring practice was still evolving in the municipalities (and, properly speaking, will never be fully stabilized), it is doubtful whether it would emerge among the hospitals and GPs if the time frame were extended. 

A common limitation concerning qualitative studies relates to generalization to broader populations. In this study, two different methods for generalization in qualitative studies can be utilized. First, the qualitative case study enables analytical generalization, performed through the connection between the case and the theoretical framework (i.e., the emergence and unfolding of practices in an inter-organizational setting in this study) [36]. Second, to enhance the transferability of the results, in-depth descriptions of the telemonitoring program, the organizational settings, and context are provided [37]. Accordingly, these results may be transferred to similar inter-organizational settings where the organizations have complementary capabilities and are dependent on each other to operate a shared telemonitoring program. However, more studies of telemonitoring in inter-organizational settings would enhance our understanding of how telemonitoring is aligned within and across organizations. Particularly, it would be interesting with studies were hospitals or GPs were main actors in delivering telemonitoring services in collaboration with community care organizations. 

## 11. Conclusions

Telemonitoring is conceived as a promising and efficient way to deliver more integrated healthcare services directly at the patients’ home. Great expectations of telemonitoring have resulted in extensive experimentation with various telemonitoring services around the world. However, positive results from pilot studies are difficult to replicate when implemented on a larger scale [4,6]. An explanation of the absence of effect can be found by focusing on the telemonitoring practices that emerge and unfold as telemonitoring is adopted, modified, or ignored in the mundane activities performed by the health professionals. From a practice-based approach, this study reveals how telemonitoring emerges and unfolds rather differently in an inter-organizational setting with municipalities, hospitals, and GPs. Although the telemonitoring technology was laden with inscriptions to guide behavior, activities, and knowledge, telemonitoring was enacted divergently, from integration into existing work practices to ignorance of telemonitoring. This divergence may be explained by the different actors’ power to adopt, adjust, or ignore such novel practices. However, this uneven enactment of the telemonitoring program led to a disconnection of practices across health providers and made it impossible to reach the shared goals for the program. Findings from this study contribute to explaining the lack of effect when implementing telemonitoring on a large scale and in complex inter-organizational settings. Conclusively, managers and health policy-makers must take into account how telemonitoring reconfigures existing practices and relations between various health professionals from divergent healthcare organizations. Alignment with existing practices *within* organizations and *across* organizations is crucial to connect the various practices and to make telemonitoring work in a complex inter-organizational setting.

## Figures and Tables

**Table 1:** Main characteristics of practices and approaches to COPD patients.

Characteristics	Municipalities	GPs	Hospitals
Practice	Care practice	Medical practice	Clinical practice
Approach to COPD patients	Holistic	Holistic Individualized	Limited to diagnosis
Knowledge	Generalized knowledge about care Use of ‘soft’ knowledge about patients’ life and overall wellbeing	Generalized medical knowledge Use of ‘soft’ knowledge about patients’ overall medical history and wellbeing	Specialized knowledge Use of ‘hard’ evidence in terms of vital signs
Organization of work	Non-acute Day-time shifts Planned work	Non-acute Day-time shifts Planned work	Acute 24-h treatment Planned and non-planned work
Autonomy in work	Low autonomy No medical responsibility	High autonomy Medical responsibility	High autonomy Medical responsibility
Dependencies among the actors due to the program	Dependent on medical authorities, i.e., GPs and hospital physicians Dependent on GPs’ accessibility and willingness to collaborate	Dependent on municipal actors’ competencies to assess patients	Dependent on municipal actors knowledge about the patients
Intended change	Using telemonitoring as primary contact to the patients Larger role in treatment and care of COPD patients to prevent hospitalizations	Adjusting patients’ treatment based on monitoring data (or the municipal actors interpretation of monitoring data) Using monitoring data in relation to consultations with patients	Substituting or reducing existing activities with telemonitoring Using the municipal actors’ knowledge about patients’ conditions

Note: COPD = chronic obstructive pulmonary disease; GP = general practitioner.

**Table 2:** Data sources.

Data Sources	Actors/Activities
Interviews (repeated after 12 months)	2 district nurses, municipality
	3 health center nurses, municipality
	2 nurses, hospital
	2 lung physicians, hospital
	6 GPs
Observations	2 district nurses’ work practices related to telemonitoring
	3 health center nurses’ work practices related to telemonitoring
	2 hospital nurses’ work practices related to telemonitoring
	4 meetings in an inter-organizational implementation group for local project managers
	1 meeting in an inter-organizational and cross-disciplinary health group in the program
Documents	Formalized descriptions of tasks, roles, functions, division of tasks and responsibilities, work instructions in relation to the telemonitoring program, minutes from meetings, business case for the program, local and national healthcare strategies
